# Anaerobes in Late-Onset Prosthetic Joint Infection (PJI) and Colorectal Carcinoma

**DOI:** 10.3390/jcm15082870

**Published:** 2026-04-10

**Authors:** Shi Ting Chiu, Mann Hong Tan, Seo Kiat Goh, Audrey Xinyun Han, Hee Nee Pang, Seng Jin Yeo, Sheng Xu, Eric Liu Xuan

**Affiliations:** Department of Orthopaedic Surgery, Singapore General Hospital, Singapore 169608, Singapore

**Keywords:** prosthetic joint infection, anaerobes, colorectal carcinoma, late onset prosthetic joint infection, case series

## Abstract

**Background:** Late-onset anaerobic prosthetic joint infection (PJI) is uncommon but may indicate underlying, previously asymptomatic colorectal malignancy. While the association between *Streptococcus bovis* group (SBG) bacteremia and colorectal cancer is well established, links between anaerobic PJIs and colorectal neoplasia are rarely reported. Anaerobic organisms originating from the gastrointestinal tract may translocate via the hematogenous route, and their presence in PJI should prompt clinicians to consider occult colorectal pathology. **Methods:** All periprosthetic arthroplasty infection cases between 2015 and 2025 were reviewed. Clinical records, diagnostic findings, microbiological data, and treatment outcomes were analyzed. **Results:** Three female patients (mean age 76.3 years) presented with late-onset PJI occurring at least five years after primary total knee arthroplasty. Causative organisms included *Bacteroides fragilis*, *Morganella morganii*, and *Klebsiella pneumoniae*. All patients underwent two single-stage revision surgeries and one debridement, antibiotics and implant retention (DAIR) procedure. Cross-sectional computed tomography imaging of the abdomen and pelvis (CT-AP) performed to evaluate hematogenous sources of infection consistently revealed previously undiagnosed colorectal malignancy. One patient had additional metastatic disease. Postoperative complications included one case of pulmonary embolism; no other major complications were observed. **Conclusions:** Anaerobic PJIs are rare, and their association with colorectal malignancy is not well established. These cases highlight the importance of evaluating potential gastrointestinal sources, including occult colorectal cancer, in patients presenting with late-onset anaerobic PJI.

## 1. Introduction

Prosthetic joint infection (PJI) is a serious complication following total knee arthroplasty (TKA), often necessitating complex revision procedures and resulting in significant morbidity. While advances in surgical technique and perioperative care have reduced infection rates, hematogenous seeding from distant sites remains an important cause of late-onset PJI.

Anaerobic PJIs are rare but they do have several risk factors associated with it that are related to the host, surgery, and microbiologic conditions. Patient-related factors include comorbidities such as patients who are immunocompromised, have diabetes mellitus, obesity, malnutrition, chronic kidney disease, and conditions that would result in poor tissue oxygenation, all of which would favor the growth of anaerobic organisms. Surgical factors including prolonged operative time of more than 90 min, or an increased complexity of the case would also increase the risk of PJI [1]. In essence, delayed diagnosis and inadequate treatment can contribute to under-recognition and persistence of anaerobic pathogens, further increasing complication rates and morbidity in patients.

Colorectal malignancy is a recognized source of bacteremia, particularly when mucosal integrity is compromised. Organisms such as *Streptococcus bovis* and *Enterococcus faecalis* have well known associations with colorectal neoplasia. Disruption of mucosal barriers in colorectal carcinoma facilitates microbial translocation and may predispose patients to hematogenous seeding of prosthetic joints.

While the majority of PJIs are caused by aerobic Gram-positive organisms such as *Staphylococcus aureus* and coagulase-negative staphylococci, anaerobic bacteria represent a relatively rare subset of pathogens. Reported incidence rates of anaerobic PJIs vary widely depending on diagnostic techniques and study design, but are most commonly cited in the range of approximately 3–6%, with some series reporting up to 8% or higher [2].

Despite these established associations, limited literature describes anaerobic PJIs linked to colorectal cancer. Understanding the interplay between colorectal cancer and PJI has implications for both infection prevention strategies and cancer screening protocols in patients undergoing or presenting with prosthetic joint complications. However, there has been a paucity of research on anaerobic PJI and concomitant colorectal neoplasia. Anaerobes account for approximately 8% of culture-positive PJIs [3], suggesting that unrecognized gastrointestinal pathology may be more common in this subgroup than previously appreciated. This study presents three cases of anaerobic PJI associated with newly diagnosed colorectal malignancy and explores the possible pathophysiological mechanisms underlying this association.

## 2. Materials and Methods

This study was conducted as a retrospective case series at a single tertiary centre. Institutional review board approval was obtained prior to data collection (CIRB 2023/2465). All patients provided informed consent for inclusion.

Case identification was performed using a structured, multi-source search strategy to ensure comprehensive case capture. Initially, departmental morbidity and mortality records were reviewed to identify patients with documented prosthetic joint infections (PJI). This was supplemented by a systematic search of the institutional electronic medical record using pre-defined keywords, including “prosthetic joint infection,” “anaerobic,” and “colorectal carcinoma”. Operative notes from orthopaedic surgery procedures during the study period were also screened to identify additional potential cases.

We identified 124 patients diagnosed with PJI between 2015 and 2025. Among this group of patients, only three patients were diagnosed with PJI with anaerobic organisms. Inclusion criteria consisted of (1) age ≥ 18 years, (2) presence of a prosthetic joint, (3) diagnosis of PJI based on established criteria such as those defined by the International Consensus Meeting (ICM) 2018 [4], and (4) isolation of at least one anaerobic organism from aspiration joint fluid cultures. Microbiological diagnosis was based on samples obtained under aseptic techniques, including preoperative joint aspiration where feasible, and tissues samples were also obtained intra-operatively with a minimum of three to five samples. Common skin commensals isolated in a single specimen without corroborative findings were regarded as potential contaminants and excluded [5].

In the three included cases, the Infectious Diseases team was also on board for the management of PJI. Computed tomography of abdomen and pelvis (CT-AP) scan was performed as recommended by the Infectious Diseases team to identify a potential gastrointestinal source. Concomitant colorectal carcinoma was discovered in these three patients on CT-AP scans, and diagnosis of malignancy was confirmed with subsequent colonoscopy and histology.

## 3. Case Descriptions

**Figure 1:**
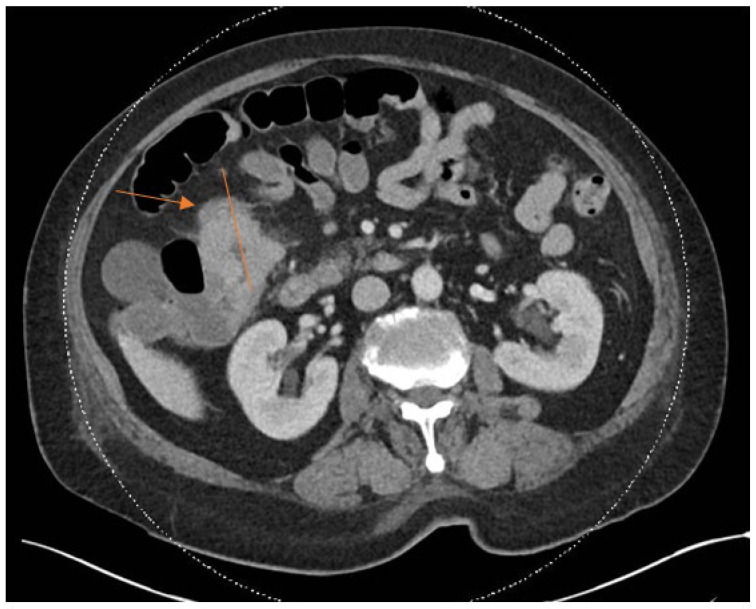
Axial cut of CT-AP scan showing colorectal carcinoma as indicated by arrow.

**Figure 2:**
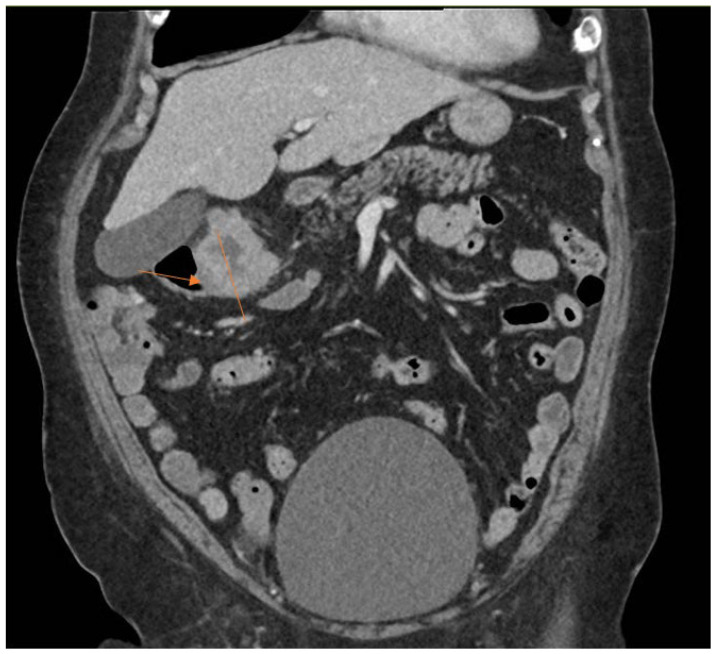
Coronal cut of CT-AP scan showing colorectal carcinoma as indicated by arrow.

The clinical characteristics of the three cases are summarized in Table 1. The laboratory characteristics of the three cases are summarized in Table 2. All three cases in our study presented with knee pain and swelling at least after five years from the primary total knee arthroplasty. Two out of three cases underwent single-stage revision surgery, whereas one case underwent the DAIR procedure. Laboratory markers indicative of acute inflammatory processes such as CRP and ESR were raised markedly in all three cases. The CT-AP scan performed in Patient 1 showed a colorectal mass, as seen in Figure 1 and Figure 2.

### 3.1. Case 1: Bacteroides fragilis PJI and Colorectal Neoplasia

A 75-year-old woman who had undergone bilateral total knee arthroplasties in 2020 presented five years after her primary total knee arthroplasties with one month of right knee pain, swelling, and a single episode of low-grade fever. The patient had no significant medical history. She denied any injury or trauma to her knees. She attended the Emergency Department once prior with the same presentation and was given analgesia and discharged after. On clinical examination, her right knee was slightly warm and erythematous with mild effusion, and her range of movement was 0 to 100 degrees. Joint aspiration performed under aseptic technique grew *Bacteroides fragilis* (*B. fragilis*). Inflammatory markers were markedly elevated (CRP 133 mg/L, ESR 131 mm/h).

She underwent a single-stage revision surgery. Intraoperative findings revealed copious purulent fluid; and intraoperative cultures confirmed *B. fragilis*. Postoperative CT-AP revealed a suspicious transverse colon mass. Subsequent colonoscopy confirmed a large proximal transverse colon tumour. Diagnosis of colorectal carcinoma was confirmed with histology showing adenocarcinoma. Computed tomography of the thorax demonstrated incidental pulmonary emboli, and anticoagulation therapy was initiated. She was discharged on long-term intravenous antibiotics and scheduled for definitive colorectal surgery after completing anticoagulation. The patient underwent a total of three months duration of antibiotics, consisting of six weeks of intravenous amoxicillin-clavulanic acid and six weeks of oral metronidazole.

The patient subsequently underwent surgical resection. At follow-up visits, she remained disease-free and her inflammatory markers showed a general downtrend with no evidence of PJI recurrence. She had also recovered back to her premorbid ambulation status.

### 3.2. Case 2: Morganella morganii PJI and Colorectal Neoplasia

The patient is a 77-year-old lady who has a past medical history of hypertension, hyperlipidemia and left breast invasive ductal carcinoma of which she underwent a simple mastectomy with axillary clearance in 2019. She had her left total knee arthroplasty done in 2008 and right total knee arthroplasty done in 2010. She was seen in the clinic 15 years after her right total knee arthroplasty for worsening left knee pain that worsened on ambulation. She was unable to ambulate and extend her knee actively. Her inflammatory markers were raised; CRP at 115 mg/L and ESR at 73 mm/h, respectively. Bedside aspiration of her left knee was performed under aseptic conditions, showing *Morganella morganii* (*M. morganii*). The culture was resistant to ampicillin and amoxicillin/clavulanic acid. She was thereafter admitted for late-onset *M. morganii* left knee PJI. The Infectious Diseases team suggested a CT-AP prior to revision surgery to rule out intra-abdominal sources of hematogenous seeding. CT-AP subsequently revealed a suspicious proximal transverse colon mass with lymphadenopathy.

She underwent a single-stage revision surgery, and intraoperative cultures confirmed *M. morganii*. Colonoscopy revealed a transverse colon tumour, and diagnosis of colon malignancy was confirmed with histology showing adenocarcinoma. The patient underwent a total of eight weeks duration of antibiotics, consisting of four weeks of intravenous cefepime and four weeks of oral ciprofloxacin. She was seen by the Colorectal Surgery team, and they had definitive plans for elective right hemicolectomy. The patient recovered successfully and was seen in follow-up consultations with improvement in her symptoms. Her inflammatory markers also showed a general downtrend.

### 3.3. Case 3: Klebsiella pneumoniae PJI and Colorectal Neoplasia

This patient is a 77-year-old female with a past medical history of hypertension, hyperlipidemia, hypothyroidism, uterine prolapse and Alzheimer’s Dementia. She underwent a right total knee arthroplasty in 2018 and left total knee arthroplasty in 2019. She was last seen in clinic in 2024, when she was five years status post her previous left total knee arthroplasty. She presented with one week duration of left knee swelling; limited range of motion and pain associated with fever. Joint aspiration was performed under aseptic technique, and fluid culture grew *Klebsiella pneumoniae.* Her inflammatory markers were raised; CRP at 89.4 mg/L and ESR at 99 mm/h.

She was subsequently admitted for late-onset *Klebsiella pneumoniae* PJI, likely from a hematogenous source. She did have symptoms of urinary tract infection in view of her history of uterine prolapse. The Infectious Diseases team started her on intravenous ceftriaxone and oral ivermectin for two days perioperatively and post operatively for one day. CT-AP was also recommended to work up the hematogenous source of *Klebsiella pneumoniae*. She underwent a left knee joint debridement and change of liner (DAIR). Intraoperative cultures confirmed *Klebsiella pneumoniae*.

CT-AP identified a large hepatic lesion initially suspected to be an abscess but later confirmed to be a metastatic mass. A rectosigmoid tumour was identified on colonoscopy and histological result showed adenocarcinoma. The patient was evaluated by the Hepatobiliary and Colorectal Surgery teams and prepared for oncologic resection. The patient was treated with six weeks duration of intravenous ceftriaxone and lifelong suppression with oral amoxicillin-clavulanic acid.

## 4. Discussion

This study is a descriptive case series with a hypothesis of anaerobic late-onset PJI and gastrointestinal translocation, thus leading to few cases of patients with concomitant colorectal carcinoma. Studies describing anaerobic PJIs and colorectal malignancy are not well documented and are rare. Anaerobic bacteria such as *Bacteroides*, *Morganella*, and *Klebsiella* commonly inhabit the gastrointestinal tract and may translocate through neoplastic or inflamed mucosa. *Bacteroides* is a strict anaerobe while *Morganella* and *Klebsiella* are facultative anaerobes. When the mucosal barrier has been breached, bacteria will be able to penetrate through the intestinal wall and disseminate into the sterile peritoneal cavity and bloodstream [6].

*Bacteroides* sp. are strictly anaerobic Gram-negative bacilli that are commensal members of the human gastrointestinal microbiota. Certain risk factors are associated with clinical infections with *Bacteroides* sp. including immunosuppression, rheumatoid arthritis and intra-abdominal pathology [7]. Gastrointestinal tumors, particularly adenocarcinomas, alter mucosal permeability and promote anaerobic overgrowth. Bacterial translocation from the gut lumen to the bloodstream may serve as a pathway for hematogenous prosthetic seeding. Many anaerobes can form dense, structured biofilms on metallic and polymeric prosthetic surfaces. Within biofilms, bacterial cells are encased in extracellular polymeric substances (EPS) that provide protection from host immune responses and antibiotics. Anaerobes thrive within these low-oxygen microenvironments, where redox potential is reduced and nutrient gradients favour their metabolism. Anaerobic bacteria also possess virulence factors such as lipoteichoic acids and short-chain fatty acid production that modulate host immune responses. Few studies have been conducted regarding *Bacteroides fragilis* PJI. A retrospective cohort study was previously done to assess the outcome of patients with *Bacteroides* PJI and to review risk factors associated with failure of therapy [7]. Between 1969 and 2012, 20 episodes of Bacteroides PJI were identified in 17 patients. The mean age of the patients in the cohort at the time of diagnosis was 55.6 years; 59% (n = 10) had knee involvement; 24% (n = 4) had diabetes mellitus; and 24% had a history of either gastrointestinal (GI) or genitourinary (GU) pathology prior to the diagnosis of PJI [4]. However, the study reported that the biomechanism of *Bacteroides fragilis* leading to PJI is not fully understood yet.

*Morganella morganii* (*M. morganii*) is a facultative anaerobic Gram-negative rod that primarily resides in the gastrointestinal tract, and it generally causes infection in individuals who are immunocompromised. It is a rare strain of bacteria that is usually associated with urinary tract infections, soft tissue abscesses, septic arthritis, psoas abscesses and osteomyelitis, especially in individuals who have risk factors such as diabetes, chronic kidney disease, chronic alcoholism, high BMI or a previous surgical intervention or trauma [8]. *M. morganii* is considered an unusual opportunistic pathogen and a rare cause of nosocomial infection. It is essential to treat *M. morganii* PJI with surgical intervention and long-term antibiotics suppression, as morbidity can be high in these patients. They have the ability to form biofilms and an inherent antimicrobial resistance—*M. morganii* strains produce inducible AmpC β-lactamases, thus making them resistant to ampicillin and first generation cephalosporins [9]. Hence, early detection and antimicrobial susceptibility testing is essential to avoid antimicrobial resistance, and it is an important step for successful treatment of *M. morganii* PJI. In the rare case of *M. morganii* PJI and colorectal cancer, bacterial translocation from the gut lumen to the bloodstream may serve as a pathway for hematogenous prosthetic seeding, and biofilm from the anaerobe can be deposited on the polymeric prosthetic surfaces.

*Klebsiella pneumoniae* is a facultative anaerobic Gram-negative rod that primarily resides in the skin flora and intestinal tract. It is part of the *Enterobacteriaceae* family, and it is usually the second leading cause of bloodstream infections caused by Gram-negative bacteria [10]. It is usually found in early-onset PJI, but PJI resulting from hematogenous cause of *Klebsiella* sp. is uncommon. *Klebsiella* sp. can form biofilms, and it enables the bacteria to escape host immune response and the action of antibiotics, hence promoting its ability to survive on prosthetic implants. In recent years, there have been increasing reports of multi-drug resistant *Klebsiella pneumoniae* and hence it would be crucial to have timely and more aggressive treatment. The literature has also shown that hospital-acquired *Klebsiella pneumoniae* hematogenous infections are more likely associated with malignancies, whereas community-acquired ones are mostly associated with diabetes mellitus and chronic liver disease [11]. Hence, this highlights the importance of early diagnosis of malignancies, especially gastrointestinal malignancy that could be concomitant with *Klebsiella pneumoniae* PJI.

As such, it is evident that many of these anaerobic bacteria have the potential to cause PJI, especially in patients with existing colorectal cancer. A common trait among the three anaerobic bacteria mentioned above appears to be the ability to escape host immune responses via various mechanisms such as biofilm formation. Given that these anaerobic bacteria are typically commensals in the gastrointestinal tract, the pathophysiology of these bacteria in colorectal neoplasia serving as a source of hematogenous infections may be like that of other bacteria that naturally reside in the gastrointestinal tract, such as *Streptococcus bovis*.

The shared gastrointestinal origin of these three organisms suggests a unifying pathogenic pathway: translocation across a disrupted mucosal barrier followed by bacteremia and subsequent seeding of prosthetic material. Colorectal cancer, in particular, may predispose patients to such events through mucosal ulceration, altered local immunity, and changes in the gut microbiome [12]. Although causality cannot be established from this small series, the findings raise the possibility that detection of anaerobic organisms in PJI—whether obligate or facultative anaerobes—should prompt consideration of an underlying gastrointestinal source.

PJI is a devastating complication following primary total knee arthroplasty that increases morbidity in most patients. Most commonly, Gram-positive bacteria and selective aerobic Gram-negative bacteria are seen in the cultures of a majority of patients with PJI. Making up only 3–6% of PJI cases, anaerobic PJIs are rarer and tend to cause significant complications [13]. In our study, anaerobic PJI made up 2.42% of PJI cases; hence, timely discovery and diagnosis of colorectal malignancy is crucial in these cases. Failure to do so may lead to poorer clinical outcomes, causing increased costs and healthcare burden. Thompson et al. stated that out of eight cases of PJI with *Streptococcus bovis* infection, only one was diagnosed with colorectal carcinoma [14]. PJI with *Streptococcus bovis* is known to have a well-linked association with colorectal carcinoma [15]. In our study, all three anaerobic PJI cases were diagnosed with colorectal carcinoma, and although this was limited by a small sample size, it is essential to be vigilant on timely discovery of possible colorectal carcinoma in anaerobic PJI cases.

The cases presented support the hypothesis that anaerobic PJI may serve as a clinical indicator of undiagnosed colorectal malignancy. Although causality cannot be confirmed, the consistent discovery of colorectal cancer in all three patients underscores the need for heightened clinical suspicion. Anaerobic infection may precede, coincide with, or follow cancer diagnosis. While routine investigation for colorectal malignancy is not currently recommended in all cases of PJI, a more selective approach may be warranted in patients with infections caused by anaerobic organisms without an obvious source. In the absence of longitudinal data, it remains unclear whether anaerobic PJI serves as a direct manifestation, a consequence, or merely a marker of underlying malignancy-related immunosuppression or mucosal disruption.

## 5. Limitations

This study is limited by small sample size and the retrospective nature of case collection. There is also a possibility that the observed relationship between anaerobic PJI and underlying colorectal carcinoma may be coincidental, especially given the limited sample size. As a small retrospective case series from a single centre, it is subject to selection bias and information bias. The identification of only three cases precludes any assessment of incidence, risk factors, or causal relationships. Additionally, the absence of a control group limits the ability to determine whether the observed association with colorectal malignancy exceeds that expected in the aerobic PJI population. This study is also limited by the fact that *Morganella morganii* and *Klebsiella pneumoniae* are obligate anaerobes. Larger prospective cohort studies including facultative anaerobic organisms are needed to further elucidate the relationship between anaerobic PJI and colorectal malignancy.

## 6. Conclusions

Anaerobic PJIs, while rare, may be associated with underlying colorectal malignancies. Clinicians could consider early abdominal imaging and gastrointestinal evaluation prior to revision surgery when managing late-onset anaerobic PJI. Early recognition may improve outcomes by enabling timely cancer diagnosis and treatment.

## Figures and Tables

**Table 1:** Clinical characteristics of the three patients with anaerobic PJIs.

	Type of Anaerobe	Age (Years)	Gender (Male/Female)	Duration of Onset of PJI from Index Surgery (Years)	Past Medical Conditions	Symptoms	Type of Revision Surgery
Patient 1	*Bacteroides fragilis*	75	Female	5	None	Knee pain and swelling	Single-stage revision surgery
Patient 2	*Morganella morganii*	77	Female	15	Hypertension Hyperlipidemia Left breast invasive ductal carcinoma	Knee pain and swelling	Single-stage revision surgery
Patient 3	*Klebsiella pneumoniae*	77	Female	5	Hypertension Hyperlipidemia Hypothyroidism Uterine prolapse Alzheimer’s Dementia	Knee pain and swelling	Joint debridement and change in liner (DAIR)

**Table 2:** Laboratory characteristics of the three patients with anaerobic PJIs.

Type of Anaerobe	ESR (mm/h)	CRP (mg/L)
*Bacteroides fragilis*	131.0	133.0
*Morganella morganii*	73.0	115.0
*Klebsiella pneumoniae*	99.0	89.4

## Data Availability

No new data were created or analyzed in this study.
